# Recent advances in understanding TCR signaling: a synaptic perspective

**DOI:** 10.12703/r/12-25

**Published:** 2023-10-16

**Authors:** Michael L Dustin

**Affiliations:** 1 Kennedy Institute of Rheumatology, The University of Oxford, Oxford, UK

**Keywords:** Immunology, affinity, microscopy, receptors, allostery

## Abstract

The T cell receptor is a multi-subunit complex that carries out a range of recognition tasks for multiple lymphocyte types and translates recognition into signals that regulate survival, growth, differentiation, and effector functions for innate and adaptive host defense. Recent advances include the cryo-electron microscopy-based structure of the extracellular and transmembrane components of the complex, new information about coupling to intracellular partners, lateral associations in the membrane that all add to our picture of the T cell signaling machinery, and how signal termination relates to effector function. This review endeavors to integrate structural and biochemical information through the lens of the immunological synapse- the critical interface with the antigen-presenting cell.

## Introduction

The T cell receptor (TCR) is restricted in its expression to T lymphocytes, but this is hardly a restriction. T lymphocytes carry out diverse functions in innate and adaptive recognition, and this is important to keep in mind when thinking about the signaling capabilities of the TCR^1^. TCRs are made up of one of two pairs of variable subunits, one pair being between TCRα-TCRβ and the other TCRγ-TCRδ. The genes encoding the TCR mRNAs are generated by somatic gene rearrangement of germline coding segments with additional random sequence information introduced during the joining process; this leads to a huge potential repertoire for both αβ and γδ receptors^2^. This variability is the cornerstone of adaptive immunity through recognition of peptide-MHC complexes by αβ TCR but is also used to build a number of stereotyped receptor rearrangements that enable innate-like TCR αβ and TCR γδ T cells that participate in immediate responses to pathogens in parallel with other effectors of innate immunity. The TCR αβ innate-like receptors include examples that recognize lipid antigens associated with CD1d^3^, referred to as invariant NK T cells (iNKT), and B vitamin metabolites associated with MR1^4^, referred to as mucosal activated invariant T cells (MAIT). A large subset of blood T cells using variable segments γ2 and γ9 recognize cholesterol metabolites through butyrophilins^5^. These recognition processes can be superimposed on different effector programs, including cell-mediated killing and help for immune responses against intracellular, extracellular or multicellular pathogens.

Signal transduction through TCR is based on a tyrosine kinase cascade. Both TCRαβ and TCRγδ subunits have a short cytoplasmic domain with no known signaling potential. Signaling through a tyrosine kinase cascade is carried out by the invariant subunits of the complex. The variable TCR heterodimers complex with three invariant dimers with the composition CD3ε-CD3δ, CD3ε-CD3γ and CD247 (ζ-homodimer) through a well-ordered process driven by transmembrane domain interactions^6^. The invariant subunits have cytoplasmic domains with pairs of tyrosine residues spaced to provide binding sites for the non-receptor tyrosine kinase ζ-associated protein of 70 kDa (ZAP70) after phosphorylation by lymphocyte kinase (LCK)^7^. ZAP70 then phosphorylates linker of activated T cells (LAT), which recruits a number of additional adapters, phospholipase Cγ (PLCγ) and complexes containing son of sevenless (SOS) to enable downstream signaling based on cytoplasmic Ca^2+^ increase, diacylglycerol and mitogen-activated kinase cascade initiation via the small GTPase RAS^8–10^. While different types of TCR bind diverse ligands, these are uniformly recognized on the surface of other cells rather than in solution. TCR signaling, therefore takes place in the context of synapsis between the T cell and an antigen-presenting cell^11^. Recent work on how this signaling cascade is initiated and sustained in the context of an immunological synapse will be the focus of this review.

## Synapse sensing by CD45

**Figure 1.**
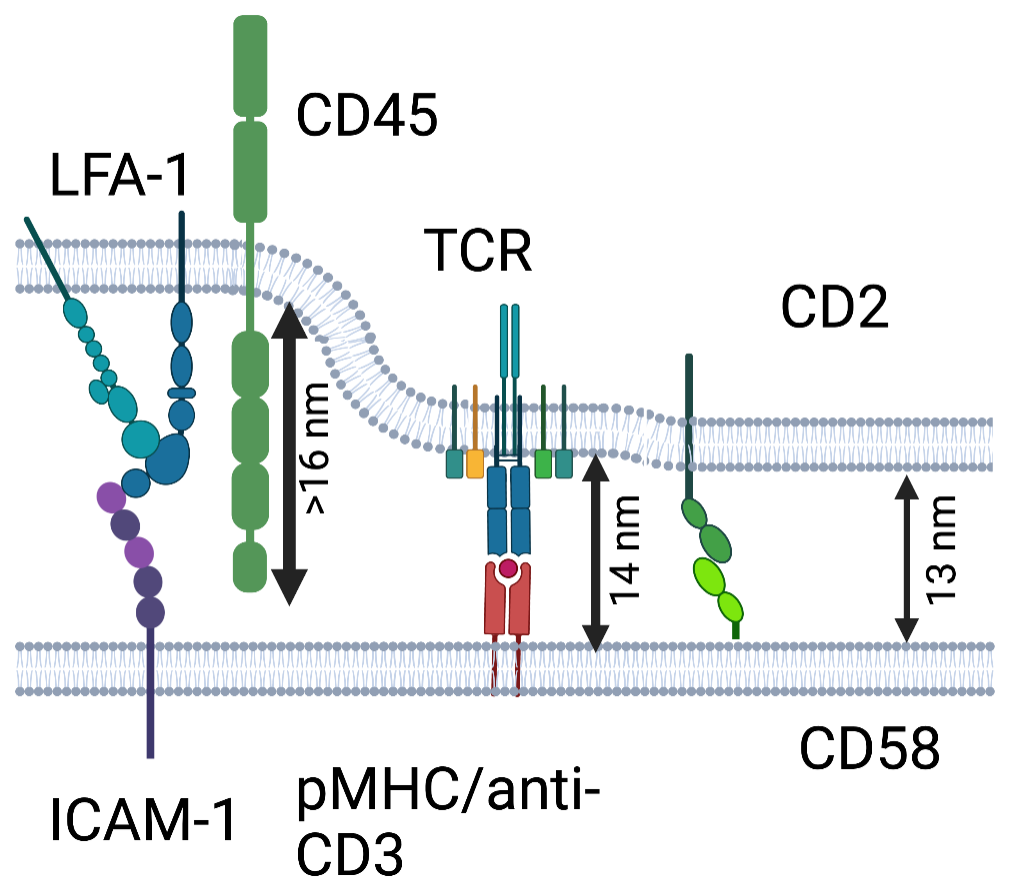
The TCR-pMHC under pressure. TCR-pMHC interaction spans ~14 nm between membranes and this is supported by the fact that this is the median distance between membranes in TCR-pMHC-mediated contacts^22^. The schematic shows this TCR-pMHC in between larger integrins and CD45 or the slightly smaller CD2-CD58 interactions. That the LFA1-ICAM1 and CD45 extracellular domains are larger than the TCR-pMHC complex is supported by lateral segregation of these interactions and the exclusion of CD45 from TCR microclusters^18^. While the TCR-pMHC and CD2-CD58 interactions could be fit into a similar intermembrane spacing and have been shown to co-localize in actin-dependent microclusters, the reduction in 2D affinity for the TCR-pMHC interaction^23^ is consistent with a small size mismatch. This may put pressure on the TCR to undergo conformational changes to fit in a closed intermembrane gap generated by CD2-CD58 trans interactions (see below about cis interactions). The figure was made in Biorender.

An important early concept for T cell antigen recognition was that the interaction of all types of TCR with ligands is typically low-affinity (with Kd values in the µM range) and requires a close approach of the T cell and antigen-presenting cells to within 13–14 nm, referred to as close contacts. This process is facilitated by adhesion molecules that reach out over greater distances and others that appear to fit into this same 13–14 nm space, leading to a proposal of a complex synapsis with at least two intermembrane spacings^12^ (Figure 1). Kupfer and colleagues captured the first images of large-scale segregation of the TCR-pMHC interactions from the larger adhesion systems like LFA1-ICAM1 in a bull’s eye pattern^13^ and studies with supported lipid bilayers revealed striking segregation of the small and large adhesion systems in interfaces formed by activated T cells^14^. The ~13–14 nm spacing is a sweet spot for optimizing the interactions of low-affinity receptors in interfaces^15^. In addition, close contacts were proposed to exclude the transmembrane tyrosine phosphatase CD45, which has a large extracellular domain > 16 nm in length^16^, facilitating activation of the tyrosine kinase cascade^12,17^, and precise CD45 exclusion was demonstrated at TCR signaling sites^18^.

Manipulation of CD45 in T cells is complicated by the essential role of CD45 to activate Lck through de-phosphorylation of the C-terminal tyrosine, which is generated through action of C-terminal src kinase (CSK)^19^. Expression of CD45 with intact phosphatase domains, but truncated extracellular domains, which would not be excluded from close contacts, has been shown to either rescue TCR signaling or inhibit it^20,21^. CD45 can be seen as a gatekeeper^19^ that promotes ligand discrimination while ensuring high sensitivity^18^. A model of CD45 exclusion from close contacts formed by TCR and other immunoreceptors combined with kinetic proofreading, referred to as kinetic segregation (KS), has become an important framework for immunoreceptor signaling across innate and adaptive immunity^24,25^. The time frames involved in kinetic segregation are on the order of a few seconds, which also represents the time frame of initial T cell triggering. Immunological synapses may last from minutes to hours, and immune responses involving innate and memory T cells can be mobilized in hours, but the first adaptive response to a pathogen, involving proliferation of rare naïve precursors, takes days to weeks to develop. CD45 is expressed on most hematopoietic cells, which thus are all equipped with a system for activation of tyrosine kinase cascades upon close contact with adjacent cells or objects - a synapse sensor!

T cells can be activated without CD45 exclusion through IS formation. For example, nanoscale engineering to generate stimulatory substrates that prevented exclusion of endogenous CD45 from sites of TCR clustering revealed that CD45 exclusion was not essential for TCR tyrosine phosphorylation if the TCR were clustered to within less than 50 nm, but the ability to exclude CD45 was required for TCR phosphorylation when TCR were > 50 nm apart^26^. Cross-linking of TCR with soluble ligands spaced less than 9 nm apart triggers T cells^27,28^. There are a number of mechanisms by which TCR clustering could lead to CD45 exclusion on a ~20 nm length scale without an IS. One possibility is lipid phase separation around clustered TCR that leads to highly localized CD45 exclusion based on mismatch of transmembrane domains^29^. It has also been noted that acute selective inhibition of CSK can lead to TCR triggering without any manipulation of CD45^30^. TCR clustering involved a phase separation process driven by interaction of the CD3ε tail with LCK. This phase separation process is self-limiting in that phosphorylation of CD3ε by LCK leads to recruitment of CSK, which favors dispersal of the TCR clusters in the absence of CD45^31^. At higher pMHC density, the clusters may become resistant to dispersal, leading to graded activation of phosphatidylinositol 3-kinase (PI3K) and ITK^32^. The ability of ITAM-based signaling to progress in the absence of CD45 exclusion is relevant in the setting of chimeric antigen receptors and bispecific T cell engagers, which can be designed to establish close contacts or greater intermembrane separation^33^. Furthermore, CSK recruitment through CD3ε phosphorylation may be less inhibitory in settings where CD45 is not as effectively excluded^34^.

## Allosteric models for the TCR

Conformational changes in the TCR have been invoked in the context of TCR engagement by ligands in the absence or presence of ~10 pN forces acting in the interface^35–37^. Catch bonds increase the duration of the TCR-pMHC interaction at ~ 10 pM force^38,39^. An interesting dilemma inherent to reconciling KS and mechanical models is that the mode of prolongation of binding can involve a significant increase in the length of the complex^36,37^, such that the binding is prolonged, but CD45 exclusion may be impaired through a local increase in membrane separation. In contrast, allosteric models for TCR signaling, in which ligand binding causes changes in conformation that are transmitted through the extracellular and transmembrane regions to change the conformation of the cytoplasmic tails, may operate in parallel with CD45 exclusion to alter the outcome of ligand binding. The cytoplasmic domains of the CD3ε and CD247, and the costimulatory CD28, all interact at rest with the inner leaflet of the plasma membrane through hydrophobic and charge-based interactions at the lipid bilayer^40,41^. These interactions are released during activation due in part to translocation of some of the acidic phospholipids to the outer leaflet of the plasma membrane^42^. Different allosteric models have been proposed, for example, based on exposure of cryptic epitopes in the receptor complex^35^, effects of small molecules that bind to the TCR on signaling^43^, or mutations that destabilize the transmembrane domain packing^44^. Conformational changes in the TCR could potentially change the local phospholipid environment, eject cholesterol, or promote dissociation of the cytoplasmic domains to become accessible to kinases and phosphatases^45,46^.

## Lessons from Cryo-structures of TCR

The structural picture of the TCR has evolved from the prior state-of-the-art crystallographic information on the interaction of variable chains of the TCR with pMHC and other ligands^47^ to a cryo-electron microscopy (cryo-EM) structure of the TCRαβ-CD3-CD247 complex in a detergent micelle^48^ and, most recently, to an engineered, high-affinity TCRαβ-CD3-CD247 complex in a detergent micelle interacting with its pMHC^49^. At face value, the earlier crystal structures and newer cryo-EM structures support a rigid body model for the TCR. In all cases, there have been minimal changes in the TCR structures with or without interaction with the pMHC. The cryo-EM structure extends this observation to the CD3 extracellular domains and the transmembrane helices. However, a recent study demonstrates a cholesterol binding-dependent change in the conformation of the transmembrane helices TCRαβ-CD3-CD247 detergent micelle complex^50^. This resonates with earlier data mentioned above that cholesterol binding helps keep the TCRαβ-CD3-CD247 complex in an inactive conformation^45,46^. Other multi-transmembrane-spanning proteins in detergent micelles display different structures than observed in phospholipid bilayers^51^. Thus, it will still be valuable to determine TCRαβ-CD3-CD247 structure in a bilayer environment with and without monovalent and multivalent ligation, in addition to collecting additional examples of TCRαβ-CD3-CD247 structures and complexes in detergent micelles. Extension to TCRγδ-CD3-CD247 would also be exciting.

In the meantime, the TCRαβ-CD3-CD247-pMHC interaction can be modeled into an interface, and molecular dynamics simulations performed to model dynamics. The TCRαβ-CD3-CD247-pMHC complex fits within a 14 nm intermembrane separation at a 30-degree angle from vertical for the TCRαβ-pMHC axis^49^. This fits with the notion that the TCR-pMHC interaction should fit into an interface stabilized by the CD2-CD58 adhesion system, as proposed earlier^12,17^. However, another recent study showed that the CD2-CD58 interaction actually decreased the 2D affinity of the TCR-pMHC in the same interface by nearly 2-fold. This suggests that the CD2-CD58 and TCR-pMHC would have slightly different intermembrane spacings^23^. Direct measurements on the CD2-CD48 interaction, which is very similar to CD58, show that it prefers a spacing of 12.8 nm^15^. Direct measurements of the intermembrane spacing in contacts dominated by TCR-pMHC averaged 13.1 nm with a modal distance of 14 nm (Figure 1). Molecular dynamics simulations of the complete TCR in a phospholipid bilayer^52^ suggest that the TCRαβ-CD3-CD247-pMHC can tilt more than observed in the cryo-EM structures to accommodate shorter intermembrane spacings. The simulations suggest that forcing the TCR to have a greater bending angle changes the conformation of the transmembrane helices, which suggests the potential for transmission of information across the membrane. The small mismatches between CD2-CD58 and TCRαβ-CD3-CD247-pMHC in the interface may also drive membrane bending that may create additional signaling opportunities^53^.

## F-actin requirements

Early studies recognized a distinct requirement for F-actin for pMHC recognition and speculated about initial contacts mediated by actin-based protrusions^54–57^, but tools to study this further were limited. Variable angle total internal reflection, expansion microscopy, and lattice light sheet microscopies have enabled closer analysis of these structures in recent years^58–61^. Studies with fixed cells show that some protrusions show concentration of TCR and partial exclusion of CD45, even before ligand binding. Live imaging suggests that many of these configurations are transient and don’t persist long enough to trigger TCR signaling. Projections with TCR enriched appear to be explained by patchiness of the TCR distribution on T cells with the F-actin-based protrusions sampling the patches randomly^61^. Approaches that generate snapshots of this dynamic situation emphasize the patchiness of receptor distribution^60,62^, whereas single-molecule approaches emphasize the transience of any observed structure and the autonomy of individual receptors over time^63^. The sources of the lateral patchiness of the T cell plasma membrane - for example, distinct domains with TCR and LAT - include lipid nanodomains^64,65^, and protein-protein interactions^62,66^. These effects may sensitize the T cell to respond upon stabilization of favorable configurations, such as CD45 segregation from the TCR, upon ligation by agonist pMHC^67^ or insertion of projections into confined spaces that lead to CD45-depleted protrusions^68^.

## Cis interactions and auto-costimulation

T cells often express combinations of costimulatory receptors and ligands that can become functionally important in cell-cell and even intracellular cis complexes. Three exemplars are based on LFA1-ICAM1, CD2-CD48/58 and CD28-CD80/86 activation of integrin LFA1 and upregulation of its ligand ICAM1 upon T cell activation results in T cell-T cell aggregation just before the cells commit to cell division and differentiation^69^. This enables collective decision-making in response to cytokines^70^. LFA1-ICAM1 interactions contribute more to T cell-T cell interaction and T cell-B cell interactions than to T cell-dendritic cell interaction^69,71,72^.

Immunoglobulin superfamily costimulatory systems have been studied assuming trans-interaction between receptors on T cells and ligands on antigen-presenting cells, although T cells often express the ligands constitutively or upon activation. Evidence for cis interactions between receptors over the past 20 years has been revisited in a number of cases recently and confirmed for costimulators CD2-CD48/58^73–75^ and CD28-CD80/86^76,77^, and negative regulators LILR2B-HLA-ABC^78^, PD1-PDL1^79^, and CTLA4-CD80/86^80^.

**Figure 2.**
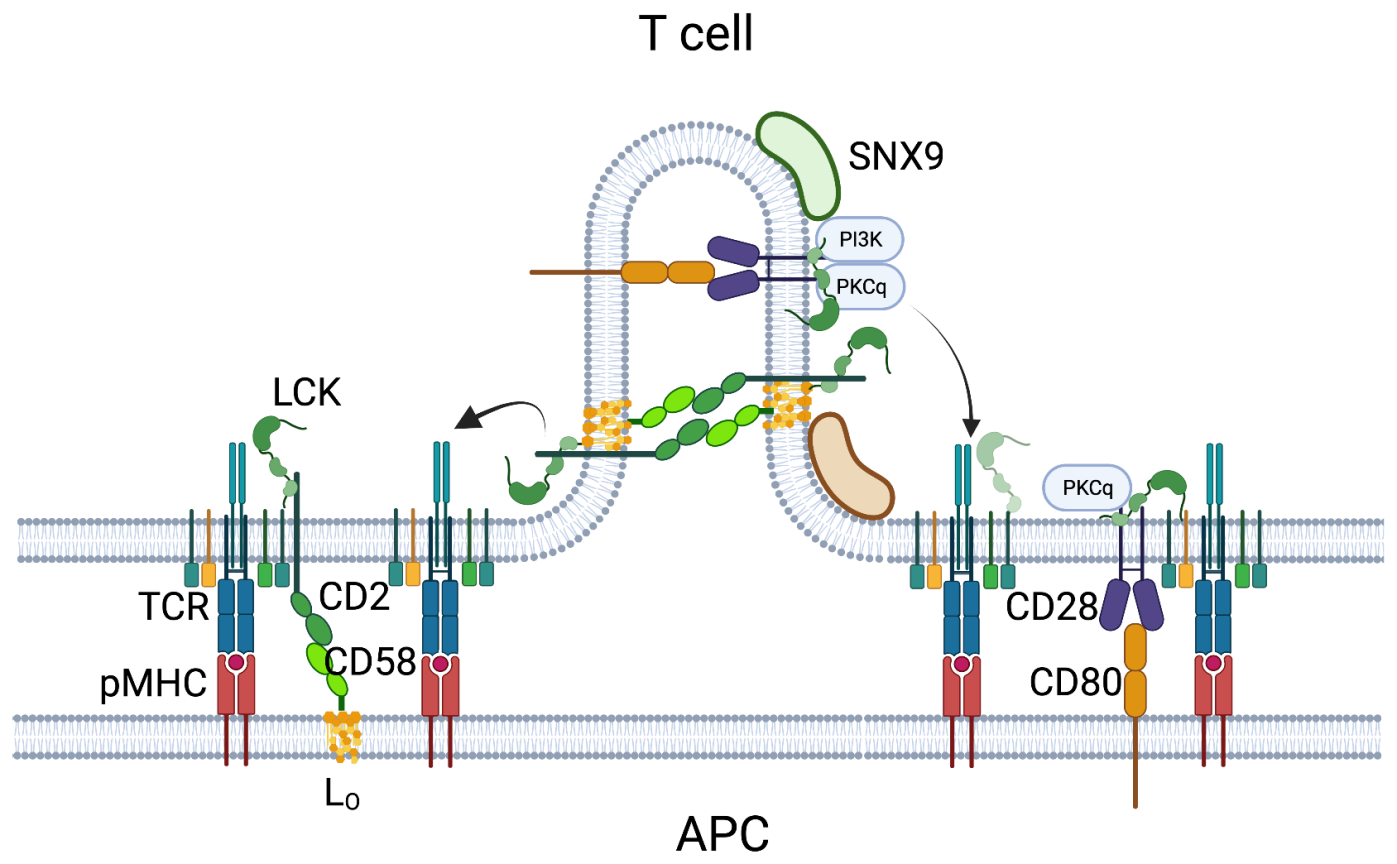
Cis costimulation amplifies TCR signals. A recent analysis of CD2 and CD48 knockout mice supported earlier reports that CD2-CD48 cis interactions inhibit adhesion mediated by CD2 interaction with CD48 in trans. However, it is not clear how CD2 and CD48, or the human ligand CD58, interact in cis. CD28-CD80/86 can also undergo cis interactions, and this has been shown to require PI3K-dependent recruitment of SNX9, leading to membrane tubule formation from the TCR/CD28 microclusters. It is within these tubules that CD28 and CD80 can interact in cis and signal for PKCθ activation and downstream signaling. We speculate here that CD2-CD58 interactions may also depend upon membrane invaginations, but this has not been demonstrated. CD48 and CD58 are GPI anchored such that they recruit liquid-ordered lipid phases (Lo) that are enriched in LCK. The size mismatch between TCR-pMHC and CD2-CD58 (or CD28-CD80) is not shown for simplicity. The figure was made in Biorender.

CD2-CD48 interactions undergo constitutive cis interactions that are detected based on competition for binding with soluble CD2-Fc or CD48-Fc comparing T cells from wild type or CD48 or CD2 knockout mice, respectively^73,75^. T cells from CD48 or CD2 knockout mice had a significantly impaired response to anti-CD3 antibodies or pMHC presented that were independent of trans-interactions with ligands on the APCs. It was proposed that CD2-CD48 cis interactions recruited liquid-ordered lipid domains to the vicinity of the active TCR^74^ (Figure 2). GPI-anchored proteins like CD48 have long acyl chains that enable transbilayer coupling to lipids in the cytoplasmic leaflet, which enables communication to lipid-anchored kinases and transmembrane proteins^81^. CD2-CD58 interaction in trans has recently been shown to organize a number of other costimulatory receptors, including CD28, ICOS, DNAM, SLAMF1, and the checkpoint receptor PD1^82^. This synapse compartment, referred to as the corolla, strongly recruited phosphorylated LAT and active LCK in human T cells engaging CD58 in supported lipid bilayers also presenting ICAM-1 and anti-CD3 or pMHC. Interestingly, when human CD2 was transiently expressed in mouse T cells, it could mediate formation of a corolla, but was unable to enhance LCK activation in the mouse T cells. Human CD2 cannot bind mouse CD48, and this suggests that the CD2 corolla’s signaling function is dependent on forming a cis network even as it binds to ligands in trans to mediate adhesion and organization of other costimulatory/checkpoint-type receptors. The CD2-CD58 interaction is highly dynamic with an off rate > 7 s^-1^^83^, so this type of cis-trans multitasking may be possible. It is not known if CD2 can interact with its ligands on a flat surface. In the case of LILRB1 cis interactions with HLA-ABC, it has been proposed that this requires bending back of the two N-terminal Ig-domains so that they can adopt a pseudo-trans configuration with respect to the pMHC^78^. It is not clear if CD2 or CD48/58 is capable of this kind of interdomain flexion. Alternatively, the cis interactions may take place in sites with negative membrane curvature, as proposed recently for CD28-CD80/86 interactions.

Auto-costimulation by CD28-CD80 interactions was reported in the context of T cell over-expression of CD80^76^, but the requirements for this process were not studied in detail. CD80/CD86 expression is low in naïve T cells, but is up-regulated upon T cell activation. Activation of PI3K through CD28 recruits the bar domain protein SNX9, leading to formation of inward membrane tubules with an appropriate diameter to allow CD28-CD80/86 interactions that activate protein kinase C-θ across the tubule lumen^77^ (Figure 2). In this context, it is important to point out that parallel work has connected SNX9 to T cell exhaustion^84^. While SNX9 may have other roles, perhaps including CD2-CD48/58 auto-costimulation, these results suggest that auto-costimulation contributes to exhaustion in the context of chronic antigen exposure.

In humans, both CD58 and CD80/86 are not expressed on naïve T cells and are expressed following activation^85^. Thus, priming of naïve T cells may be initiated with costimulation from the APC, but exposure of previously activated T cells to agonist pMHC may recruit auto-costimulation. Sufficiently strong pMHC signals may also recruit auto-costimulation over a period of hours following initial TCR stimulation. Thus, the molecular basis of “TCR signaling” in effector and memory T cells will likely incorporate components of auto-costimulation in specialized membrane compartments.

## LCK- instigator and networker

LCK plays a critical role in initiating TCR and costimulatory signaling. LCK has four interaction motifs - the N-terminal palmitoylation sites to interact with the inner leaflet of the plasma membrane, the membrane-proximal Zn^2+^ clasp that enables association with co-receptors, the SH3 domain that mediates intra and intermolecular interactions, and the SH2 domain that binds to the inhibitory C-terminal phosphorylation and to other tyrosine-phosphorylated proteins. LCK associates efficiently with co-receptors that are laterally recruited to the TCR through interactions with pMHC, MHC class II for CD4 and MHC class I for CD8. It has been suggested that CD8 may help to constrain the orientation of TCR binding to pMHC, as only the canonical orientation positions LCK for phosphorylation of the CD3 and CD247 tails^86^. For CD4, it has been further suggested that LCK bound to CD4 has access to the cytoplasmic domain of TCR bound to adjacent self-pMHC^87^. Perhaps due to these constraints, “free” LCK, not associated with co-receptors, is also involved in initial TCR signaling^88^. LCK interacts with sequences in CD3ε through its N-terminus to facilitate phosphorylation of the CD3 and CD247 phosphotyrosine motifs that recruit ZAP-70^89,90^. LCK can additionally use its SH3 domain to bridge the TCR-CD3-CD247-ZAP-70 complexes to LAT^91^, which is important for PLCγ activation, and CD28 to protein kinase C-θ^92^, which is important for AP-1 and NF-kB activation. These activities have generally been described in the context of CD2-CD48/CD58 cis interactions, which may help optimize local lipid organization and LCK availability.

## Termination and re-birth

**Figure 3.**
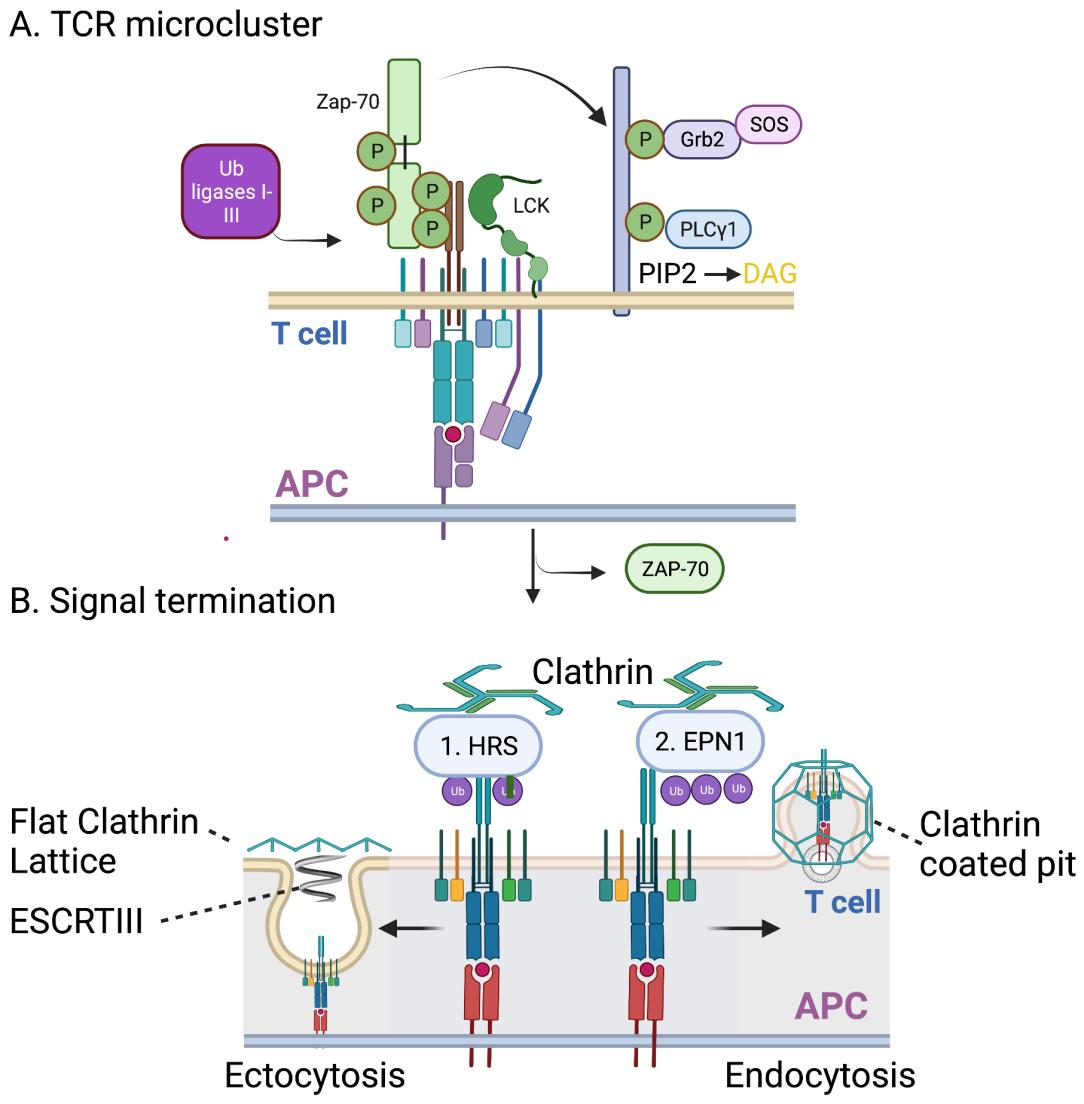
Role of opposite membrane trafficking in signal termination. **A**. Schematic of signaling from TCR to recruitment of PLCγ1 and conversion of phosphatidylinositol—4,5-bisphosphate (PIP2) to diacylglycerol (DAG). The accumulation of DAG is required for PKCθ activation. Ubiquitin ligases E1-E3 act on the signaling complexes to add mono and multiubiquitin to the cytoplasmic domains of the TCR. Ub and TSG101 are required for signal termination^97^, marked by dissociation of ZAP-70 kinase. **B**. The ubiquitinated TCR are recognized sequentially by two clathrin adapters^99^. First, HRS, which associates with flat clathrin lattices for Clathrin and ESCRT Mediated Ectocytosis (CEMI) peaking at 5 minutes into IS formation. Subsequently, EPN1 directs TCR to clathrin-coated pits for endocytosis peaking at 15 minutes. The vesicles released from CD8 cytotoxic T cells are highly enriched in DAG, which may have a role in their generation in the context of CTL dissociation from targets^100^.

Does the central accumulation of TCR in the IS sustain signaling or terminate it? Signal termination by TCR endocytosis and either recycling or degradation has long been observed^93,94^ after a few minutes of signaling. TCR microclusters undergo a maturation process as they are transported by distinct F-actin transport networks^95^ and consolidation of TCR at the center of the interface serves to focus lipid modifications associated with central F-actin reorganization to generate a fenestrated secretory zone^96^. TCR transport within this central compartment is dependent upon the endosomal complexes required for transport (ESCRT)^97^, and some of the TCR are released into the central IS by ectocytosis^98–100^. The choice between ectocytosis to release an extracellular vesicle versus endocytosis leading to degradation or recycling is based on different clathrin adapters, HRS for ectocytosis, recruited first, and EPN1 for endocytosis, recruited later^99^ (Figure 3). In helper T cells, the TCR-positive synaptic ectosomes also bear CD40 ligand (CD40LG) in response to CD40 on the antigen-presenting surface^101,102^. CD40L is a critical signal for T cell help of dendritic cells and B cells, so the transfer of CD40L in synaptic ectosomes could be critical to deliver this signal. In the context of CD8 T cells, the release of ectosomes with a high diglyceride content has been proposed to allow release of the T cells from targets for serial killing^100^. Lipidomics analysis of extracellular vesicles from CD8^+^ T cells confirms high (20%) diglyceride content^103^, which favors hexagonal over bilayer lipid phases and may lead to unstable vesicles^104^. Consistent with this, at 90 minutes after initiation of IS formation by CD8^+^ CTL, no lipid bilayer-based vesicles were recovered from the target side of the synaptic cleft, but only glycoprotein particles with a shell of thrombospondins containing cytotoxic proteins^105^. Lipid vesicles were recovered only when FAS was incorporated into the target membrane, which captures vesicles containing FASLG^105,106^. Thus, TCR signaling is terminated through a process of ectocytosis, but this same process gives birth to important effectors and serial killing.

## Conclusion

T cells not only need to recognize pMHC or other ligands with high sensitivity but must be able to discriminate between similar ligands to, for example, recognize a mutated self-protein in a tumor or autoimmunity triggered by pathogen-associated proteins that are similar to self. Similar problems are faced by TCRαβ and TCRγδ used for innate recognition, namely that the TCR platform may have evolved into innate recognition roles when the self and pathogen-associated patterns are closely related, leading to requirement for greater discrimination than in other pattern recognition systems. Recently, discrimination has been quantified as a single parameter that captures the trade-off between sensitivity and binary decisions^107^. Another recent study looked at multiple inputs and outputs with analysis of time-course data through a neural network and concluded that two parameters were needed to quantify T cell ligand discrimination^108^. Discrimination can be encoded in receptors, but also in the downstream signaling networks. For example, thymocytes are more sensitive than mature T cells as they need to be positively selected by self-pMHC, which mature T cells then ignore. At least part of the signaling program responsible for this shift is coordinated by microRNAs in thymocytes that down-regulate proteins that dampen signaling in mature T cells^109^. Memory T cells and innate-like T cells may even further attenuate signaling pathways to balance their higher abundance^110^, and use the highly sensitive TCR as a pattern recognition receptor^1^. More work is needed to integrate the contributions of CD45 exclusion, TCR allostery, auto-costimulation and signal termination to quantitative aspects of discrimination in the context of boosting natural T cell activation and T cell engineering to cope with challenges of infection, cancer and autoimmunity.
